# Angiotensin II‐induced endothelial dysfunction: Impact of sex, genetic background, and rho kinase

**DOI:** 10.14814/phy2.15336

**Published:** 2022-06-09

**Authors:** Dale A. Kinzenbaw, Lucy Langmack, Frank M. Faraci

**Affiliations:** ^1^ Departments of Internal Medicine Francois M. Abboud Cardiovascular Center The University of Iowa Carver College of Medicine Iowa City Iowa USA; ^2^ Departments of Neuroscience and Pharmacology The University of Iowa Carver College of Medicine Iowa City Iowa USA

**Keywords:** carotid artery, endothelium‐dependent vasodilation, ROCK

## Abstract

The renin‐angiotensin system (RAS) contributes to vascular disease with multiple cardiovascular risk factors including hypertension. As a major effector within the RAS, angiotensin II (Ang II) activates diverse signaling mechanisms that affect vascular biology. Despite the impact of such vascular pathophysiology, our understanding of the effects of Ang II in relation to the function of endothelial cells is incomplete. Because genetic background and biological sex can be determinants of vascular disease, we performed studies examining the direct effects of Ang II using carotid arteries from male and female mice on two genetic backgrounds, C57BL/6J and FVB/NJ. Although FVB/NJ mice are much less susceptible to atherosclerosis than C57BL/6J, the effects of Ang II on endothelial cells in FVB/NJ are poorly defined. Overnight incubation of isolated arteries with Ang II (10 nmol/L), impaired endothelial function in both strains and sexes by approximately one‐half (*p* < 0.05). To examine the potential mechanistic contribution of Rho kinase (ROCK), we treated arteries with SLX‐2119, an inhibitor with high selectivity for ROCK2. In both male and female mice of both strains, SLX‐2119 largely restored endothelial function to normal, compared to vessels treated with vehicle. Thus, Ang II‐induced endothelial dysfunction was observed in both FVB/NJ and C57BL/6J mice. This effect was sex‐independent. In all groups, effects of Ang II were reversed by inhibition of ROCK2 with SLX‐2119. These studies provide the first evidence that ROCK2 may be a key contributor to Ang II‐induced endothelial dysfunction in both sexes and in mouse strains that differ in relation to other major aspects of vascular disease.

## Graphical abstract

Because genetic background and biological sex can be determinants of vascular disease, we studied effects of angiotensin II (Ang II) on carotid arteries from mice on two genetic backgrounds (C57BL/6J and FVB/NJ). Treatment of arteries with Ang II impaired endothelial function in both strains and in both sexes by approximately one‐half. In both male and female mice on both strains, an inhibitor of Rho kinase 2 largely restored endothelial function to normal. These studies provide the first evidence that ROCK2 may be a key contributor to Ang II‐induced endothelial dysfunction in both sexes and in mice on diverse genetic backgrounds.

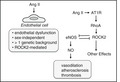

## 1 INTRODUCTION

Vascular disease, particularly atherosclerosis, is the most common cause of myocardial infarction and stroke (Bjorkegren & Lusis, 2022; Tsao et al., 2022). In relation to stroke, disease of the carotid arteries and intracranial arteries are the leading causes of ischemic stroke (Hu et al., 2017; Gasbarrino et al., 2022). The renin‐angiotensin system (RAS) is thought to be an important contributor to mechanisms by which cardiovascular risk factors promote vascular disease. As a primary effector within the RAS, angiotensin II (Ang II) exhibits pleiotropic effects including vasoconstriction and activation of pro‐oxidant and pro‐inflammatory signaling pathways that affect the function of endothelial cells and other aspects of vascular biology (Daugherty et al., 2004; Didion et al., 2005; Girouard et al., 2007; Johnson et al., 2013; Karnik et al., 2015). Defects in nitric oxide (NO) signaling are one of several causes of endothelial dysfunction involved in initiating the onset and progression of atherosclerosis (Bjorkegren & Lusis, 2022). Despite an array of changes that occur during pathophysiology, our understanding of the effects of Ang II on the vasculature and endothelial cells at the mechanistic level is still incomplete.

One target of Ang II and Ang II type 1 receptors (AT1R) is Rho kinase (ROCK), which can produce endothelial dysfunction via inhibitory effects on endothelial NO synthase (eNOS) and other mechanisms (Amin et al., 2013; Budzyn et al., 2006; De Silva et al., 2016, 2017, 2018, 2018; Sawada & Liao, 2014). While there are two isoforms of ROCK (Sawada & Liao, 2014), little is known regarding the impact of individual isoforms in relation to endothelial function in health or disease. Thus, our first goal was to evaluate the role of ROCK in Ang II‐induced impairment of endothelial function. Because carotid arteries are an important sight of atherosclerosis formation and carotid artery disease is a key contributor to ischemic stroke (Gasbarrino et al., 2022), we studied endothelial function in carotid arteries in the present study.

Genetics and genetic background are determinants of endothelial dysfunction and atherosclerosis (Bjorkegren & Lusis, 2022; Daugherty et al., 2004; De Silva, Modrick, et al., 2018; von Scheidt et al., 2017; Sontag et al., 2014). However, relatively little is known regarding the impact of genetic background in relation to endothelial dysfunction or mechanisms involved. Thus, our second goal was to examine direct effects of Ang II on carotid arteries from mice on two genetic backgrounds, C57BL/6 and FVB/N. We chose these two genetic strains because the AT1R promotes endothelial dysfunction and atherosclerosis (Daugherty et al., 2004), but FVB/N mice are much less susceptible to atherosclerosis compared to C57BL/6 mice (Sontag et al., 2014).

Cardiovascular effects of Ang II sometimes exhibit sex‐dependent differences that can be greater in males (Girouard et al., 2008; Sullivan, 2015). To gain futher insight into this issue, our third goal was to determine if direct effects of Ang II on carotid arteries were sex‐dependent.

We found that Ang II produced similar endothelial dysfunction in carotid arteries from FVB/NJ or C57BL/6J mice. These effects were sex‐independent. In all groups, effects of Ang II were reversed by SLX‐2119, an inhibitor with high selectivity for the ROCK2 isoform of ROCK. Thus, these studies provide the first evidence that ROCK2 may be a key contributor to endothelial dysfunction in both sexes and in mouse strains that differ in relation to other major aspects of vascular disease.

## 2 MATERIALS AND METHODS

The data that support the findings of this study are available from the corresponding author on reasonable request.

### 2.1 Experimental animals

The protocol was approved by the University of Iowa Animal Care and Use Committee. We studied male and female C57BL/6J and FVB/NJ (from the Jackson Laboratories) mice fed standard chow (Teklad 7913) and water *ad libitum*. The care and use of mice met the standards set by the National Institutes of Health for experimental animals. Details regarding the experimental procedures are described below. Sex differences in this initial study were evaluated using adult male and female mice with intact gonads (Morselli et al., 2016). Studies were performed in 2018 and 2019.

### 2.2 Carotid artery function

Following euthanasia with isoflurane, carotid arteries were removed, placed in Krebs buffer, and loose connective tissue was removed. Vascular segments were then cut into rings (3–4 mm in length), Vascular rings were then placed in culture wells at 37 °C for 22 hrs. Details regarding the culture media and approach are described elsewhere (Didion et al., 2005). Individual wells were treated with vehicle (saline) or Ang II (Didion et al., 2005).

When we first considered using this model, we examined findings in the literature (e.g., Berry et al., 2000; Jung et al., 2004), demonstrating that effects of Ang II were basically the product of the concentration of Ang II and time of treatment (Berry et al., 2000). With longer periods of time, lower concentrations of Ang II could be used. In contrast, higher concentrations of Ang II could produce effects within a few hours. Because we wanted to avoid using high concentrations of Ang II, we combined the use of relatively low concentrations of Ang II with overnight (22 hours) incubation. With that design, we found that Ang II (1–10 nmol/L) produced concentration‐dependent endothelial dysfunction in carotid arteries from male C57BL/6 mice (Chrissobolis et al., 2008; Didion et al., 2005), and that the response of arteries incubated overnight with vehicle versus fresh vessels to endothelium‐dependent agonists were very similar (Didion et al., 2005; Schrader et al., 2007). Thus, we used that same concentration of Ang II in the current experiments. This basic model has been used in other laboratories, including experiments using other agonists to produce endothelial dysfunction (Cascino et al., 2011; Freed et al., 2014; Itani et al., 2016; Mukohda et al., 2016; Schrader et al., 2007).

Following overnight incubation, arteries were suspended in organ baths to measure isometric tension (contraction or relaxation). After a stabilization period, vessels were contracted (50–60% of maximum) using U46619. Acetylcholine was used to test endothelium‐dependent relaxation and nitroprusside for endothelium‐independent relaxation. We found previously that responses to acetylcholine in carotid arteries from both male and female mice are mediated by eNOS (Lamping & Faraci, 2001). Other laboratories reported similar results in carotid arteries of mice and cerebral arteries of humans (Chataigneau et al., 1999; d'Uscio et al., 2001; Lamping & Faraci, 2001; Scotland et al., 2002; Segarra et al., 1999). SLX‐2119 (1 μmol/L) was used to determine if vascular dysfunction was mediated by ROCK (e.g., ROCK2). We found previously that this concentration of SLX‐2119 is effective is reversing inhibitory effects of an activator of RhoA on endothelial function in these same arteries (De Silva et al., 2016).

### 2.3 Drugs

Acetylcholine, angiotensin II, and nitroprusside were obtained from Sigma. U46619 (Cayman Chemical) was dissolved in ethanol with subsequent dilutions made in saline. SLX‐2119, was purchased from ApexBio. SLX‐2119 was dissolved in 100% dimethyl sulfoxide (DMSO) at a concentration of 1 mmol/L and subsequently diluted in saline such that the final concentration of DMSO was ≤0.1%. Other drugs were dissolved and diluted in saline.

### 2.4 Statistical analysis

All data are expressed as mean ± SE. Data were evaluated using Prism 8 software and either an unpaired *t*‐test or two‐way ANOVA followed by Tukey's post‐hoc test. Details are provided in the text or figure legends. Statistical significance was accepted at *p* < 0.05.

## 3 RESULTS

### 3.1 Baseline features

Body weight was similar in male C57BL/6J (31.7 ± 1.1 g) and FVB/NJ (31.5 ± 0.6 g) mice (*p* > 0.05), but was less in female mice of both strains (25.9 ± 1.0 vs. 25.7 ± 0.6 g in C57BL/6J and FVB/NJ, respectively). Based on unpaired *t*‐tests, the differences in body weight between males and females of both genotypes [n =7 C57BL/6J (*p* = 0.0017) and *n* = 8 FVB/NJ (*p* < 0.0001)] were significant. There were no significant differences (based on unpaired *t*‐tests) between ages in male or female mice of either strain. Age (in months) was 4.4 ± 0.2 and 4.6 ± 0.4 in male and female C57BL/6J mice, respectively [n =7 (*p* = 0.740)]. Age in FVB/NJ mice was 5.4 ± 0.4 and 6.4 ± 0.8 in males and females, respectively [n =8 (*p* = 0.307)].

### 3.2 Impaired endothelial function in response to Ang II

**FIGURE 1:**
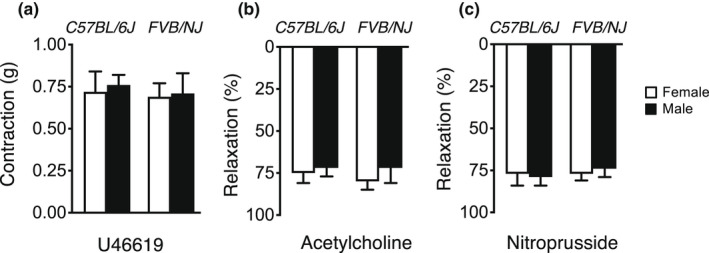
Responses to maximal concentrations of acetylcholine (10 µmol/L), nitroprusside (10 µmol/L), and U46619 (3 µg/ml) in carotid arteries from female and male C57BL/6J (*n* = 7) and FVB/NJ (*n* = 8) mice following incubation with vehicle. All data are mean ± SE.

**FIGURE 2:**
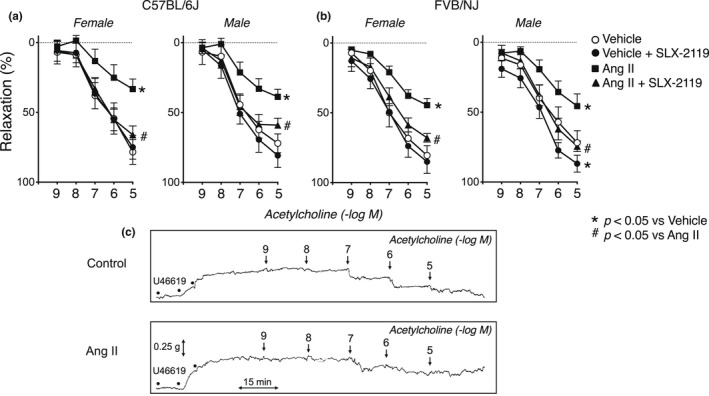
Concentration‐dependent effects of acetylcholine on carotid arteries from female and male C57BL/6J (*n* = 7) (a) and FVB/NJ (*n* = 8) mice (b) following incubation with vehicle or Ang II. All data are mean ± SE. Statistical differences were based on two‐way ANOVA followed by Tukey's post‐hoc test. *p < 0.05 versus vehicle within the same sex and genotype. ^#^ *p* < 0.05 for Ang II versus Ang II plus SLX‐2119 within the same sex and genotype. (c) Relaxation of the carotid artery in response to acetylcholine in vascular rings from a female C57BL/6J mouse after treatment with vehicle (Control) or Ang II. Vascular rings were precontracted submaximally with U46619. Concentrations of acetylcholine are shown above each tracing.

Baseline responses of arteries treated with vehicle were similar in female and male C57BL/6J and FVB/NJ mice (Figure 1). For ease of comparison, maximum effects of U46619, acetylcholine, and nitroprusside in the different groups are presented in Figure 2a–c.

Consistent with previous studies, acetylcholine produced concentration‐dependent relaxation of carotid arteries precontracted submaximally with U46619 (Figure 2a,b). Treatment with Ang II inhibited the vasodilator response to acetylcholine in both female and male C57BL/6J and FVB/NJ mice (Figure 2a,b). Inhibitory effects of Ang II on maximal responses to acetylcholine ranged from approximately 40–60%. An example of responses of carotid arteries from a female C57BL/6J mouse is shown in Figure 2c. These findings indicate that relaxation of the carotid artery to acetylcholine is impaired by local treatment with Ang II. These effects were observed in both sexes and in both C57BL/6J and FVB/NJ mouse strains.

**FIGURE 3:**
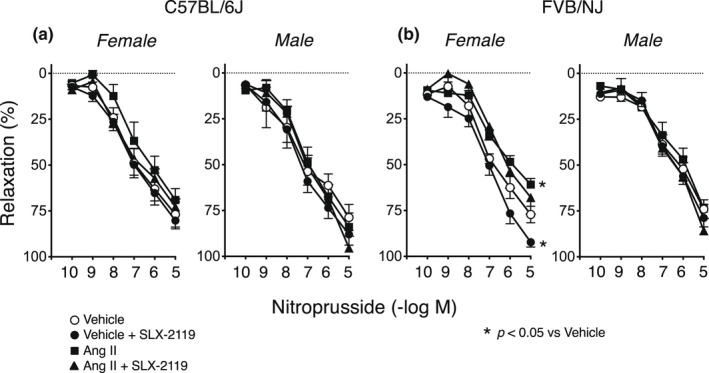
Responses of carotid arteries to nitroprusside in female and male C57BL/6J (*n* = 7) and FVB/NJ (*n* = 8) mice following incubation with vehicle or Ang II. All data are mean ± SE. Statistical differences were based on two‐way ANOVA followed by Tukey's post‐hoc test. *p < 0.05 versus vehicle within the same sex and genotype.

Baseline vascular responses to the endothelium‐independent NO donor (nitroprusside) were similar in arteries treated with vehicle from male and female mice on either genetic background (C57BL/6J or FVB/NJ) (Figure 3). In most groups, treatment with Ang II had no significant effect on relaxation of carotid arteries to nitroprusside (Figure 3). Relaxation of arteries from female FVB/NJ mice was modestly impaired by Ang II (Figure 3). Thus, the effects of Ang II treatment were predominantly endothelium‐specific.

### 3.3 Ang II‐induced endothelial dysfunction was largely eliminated by inhibition of ROCK2

Ang II‐induced endothelial dysfunction was completely or largely eliminated by acute treatment with SLX‐2119 in male and female mice of both genetic backgrounds (Figure 2). SLX‐2119 had no significant effect of responses to acetylcholine in any group treated with vehicle except for male FVB/NJ mice, where a modest enhancement was observed (Figure 2b). In contrast, SLX‐2119 increased responses to acetylcholine in arteries in all groups after treatment with Ang II. This restoration of endothelium‐dependent relaxation was similar in magnitude regardless of genetic background and was sex‐independent (Figure 2). There were no significant effects of SLX‐2119 on relaxation to the NO donor in any group with the exception of a small enhancing effect in female FVB/NJ mice (Figure 3b).

## 4 DISCUSSION

There are several findings in the present study. First, local treatment with Ang II selectively impaired endothelial function in carotid arteries. Second, vascular effects of Ang II were similar in both male and female mice on two distinct genetic backgrounds ‐ FVB/NJ and C57BL/6J. Thus, within the scope of these experiments, the effects of Ang II were sex‐ and strain‐independent. Third, effects of Ang II on endothelial function were reversed by inhibition of ROCK2 with SLX‐2119 in all groups. These studies provide the first evidence that ROCK2 may be a key contributor to endothelial dysfunction in both sexes and in mouse strains that differ in relation to other major aspects of vascular disease (e.g., atherosclerosis). Overall, our experiments provide new insight into phenotypic effects of Ang II on arteries and the mechanistic impact of ROCK2.

### 4.1 Effects of Ang II on endothelial function

Consistent with previous studies, Ang II produced endothelial dysfunction in the current experiments. An advantage of the model used is the testing of direct effects of Ang II on the vessel wall without confounders such as differences in arterial pressure that occur in some models when Ang II is administered systemically (Sullivan, 2015). The current model phenocopies effects of chronic systemic treatment with Ang II on endothelial function (Johnson et al., 2013; Li et al., 2016; Schrader et al., 2007). The focus on endothelial cells is appropriate as endothelial health is a determinant of vascular disease and overall longevity (Bjorkegren & Lusis, 2022; De Silva & Faraci, 2020; Sun et al., 2020).

Vascular effects of Ang II, as seen in the present study, are not specific for the carotid artery. Impairment of vascular function in response to Ang II has been described in aorta and cerebral arteries (although higher concentrations of Ang II were used). (Jung et al., 2004; Leo et al., 2015) In relation to microvessels, some work in this area has also been done. Small human or porcine arteries have been isolated and incubated with ceramide or Ang II (respectively) in vitro, followed by studies of endothelial function (Freed et al., 2014; Zhang et al., 2003). Thus, studies of smaller vessels using this model are feasible.

Available data suggests endothelial dysfunction is not unique to arteries treated with Ang II or a response to vasoconstriction. Although Ang II is a potent vasoconstrictor in some vascular beds, it is a very weak constrictor of the mouse carotid artery (Chiossi et al., 2011; Meyer et al., 2014). In addition, select vasodilators including lipopolysaccharide, interleukin‐1ß, and ceramide can also produce endothelial dysfunction (Didion & Faraci, 2005; Didion et al., 2004; Freed et al., 2014; Mukohda et al., 2016). Thus, vasoconstriction per se is not required to produce endothelial dysfunction.

### 4.2 Sex‐dependent effects of Ang II

Baseline responses of carotid arteries to acetylcholine and nitroprusside, as well as contraction to U46619 were similar in male and female mice, findings that are similar to results from previous studies (De Silva et al., 2017; De Silva, Li, et al., 2018; Faraci et al., 2006). As suggested by experts in the area, experiments in this initial study were performed using adult male and female mice with intact gonads (Morselli et al., 2016).

Some cardiovascular effects of Ang II are sex‐dependent (Faraci et al., 2006; Girouard et al., 2008; Sullivan, 2015). In the current study, we found that direct effects of Ang II on endothelial function was not sex‐dependent. Although such findings may seem somewhat surprising, there are studies in humans and preclinical models where the effects of Ang II were sex‐independent, and other studies where Ang II caused greater vascular responses in women than in men (Gandhi et al., 1998; Mendonca et al., 2014; Schneider et al., 2010). Thus, a single pattern is not applicable across all studies.

### 4.3 Impact of genetic background on Ang II‐induced endothelial dysfunction

Effects of Ang II on the vasculature has been studied predominantly using male C57BL/6 mice. A few studies have performed similar experiments using mice on other genetic backgrounds. For example, systemic treatment of FVB/N or BALB/c mice with Ang II selectively impaired endothelium‐dependent vasodilation to acetylcholine in aorta and mesenteric arteries (Virdis et al., 2004; Widder et al., 2007), findings that are consistent with the current observations.

Genetic variation, or differences in genetic background, can have a major influence on vascular biology (Bjorkegren & Lusis, 2022; De Silva, Modrick, et al., 2018; von Scheidt et al., 2017; Yang et al., 2021). Rather than basing all conclusions on a single genetic strain, we used mice on two very different genetic backgrounds. Genetic background affects vascular biology, vascular aging, and development of atherosclerosis (Bjorkegren & Lusis, 2022; von Scheidt et al., 2017), the latter is a leading risk factor for ischemic stroke and likely contributor to dementias (De Silva, Modrick, et al., 2018; Gasbarrino et al., 2022; von Scheidt et al., 2017).

In the current study, we found that direct effects of Ang II on endothelial function was similar in C57BL/6J and FVB/NJ mice. These results differ from previous findings where aging reduced endothelial function in the same two strains of mice, but the magnitude of reduction was greater in C57BL/6 compared to FVB/N mice (De Silva, Modrick, et al., 2018). In all groups, effects of Ang II were reversed by inhibition of ROCK2 with SLX‐2119 (De Silva, Modrick, et al., 2018).

### 4.4 Impact of ROCK

Ang II‐dependent changes in vascular function have been studied widely, and have implicated key contributions by multiple mechanisms including reactive oxygen species (ROS) and oxidative stress (Berry et al., 2000; Didion et al., 2005; Johnson et al., 2013; Karnik et al., 2015; Reckelhoff & Romero, 2003; Schrader et al., 2007). Interactions between ROS and ROCK have been described. For example, ROS activates RhoA via effects on a redox sensitive motif (Aghajanian et al., 2009), followed by ROCK activation (Kahles et al., 2007; Schreibelt et al., 2007). Peroxynitrite, the chemical product of NO reacting with superoxide, also activates RhoA and ROCK in endothelial cells (Choi et al., 2018). Fasudil inhibits the expression of NADPH oxidase components and the formation of superoxide in aorta in a model of Ang II‐dependent hypertension (Higashi et al., 2003).

For the current study, we wanted to examine potential interacting mechanisms in more detail. One of the consequences of increased ROS is activation of ROCK, but insight into the role of specific ROCK isoforms (ROCK1 vs. ROCK2) in relation to endothelial function is very limited. Thus, we used SLX‐2119 in the current study to partially fill this knowledge gap. SLX‐2119 is highly selective for ROCK2 compared with ROCK1 (Ki: >10,000 nmol/L for ROCK1 vs. 41 nmol/L for ROCK2) (Lee et al., 2014). In the current experiments, treatment of arteries with Ang II impaired endothelial function by approximately one‐half. To examine the potential contribution of ROCK2, we treated arteries with SLX‐2119. In males and females of both strains, SLX‐2119 restored endothelial function toward normal. SLX‐2119 had little effect on vascular responses in the absence of Ang II.

Cell‐specific genetic interference with PPARγ in endothelial cells increases vascular sensitivity to Ang II, and effect that occurs in both male and female mice but is reversed by either Y‐27632 (an inhibitor of ROCK1 and ROCK2) or SLX‐2119 (De Silva et al., 2017). In aged mice with endothelial‐specific genetic interference with PPARγ, Y‐27632 did not affect vascular responses in adult controls, but did restore endothelial function in the genetically‐altered mice (De Silva, Li, et al., 2018). These effects were similar in males and females.

Although two isoforms of ROCK exist, a lack of available genetic models or isoform‐specific inhibitors have limited insight into the impact of ROCK1 versus ROCK2 in vascular cells and models of vascular disease. The commonly used ROCK inhibitor Y‐27632 has similar inhibitory constants for both ROCK1 and ROCK2 (De Silva, Modrick, et al., 2018). In contrast, SLX‐2119 has high selectivity for ROCK2 (De Silva et al., 2016, 2017). In recent studies, both Y‐27632 and SLX‐2119 had similar effects on vascular function in several models, suggesting that ROCK2 was the isoform primarily responsible for endothelial dysfunction (De Silva et al., 2016, 2017; De Silva, Modrick, et al., 2018). These observations, along with a lack of selective pharmacological inhibitors of ROCK1, prompted us to focus on ROCK2 in the current study. Consistent with previous work (De Silva et al., 2016, 2017), we found that Ang II‐induced endothelial dysfunction (in both sexes and in mice on different genetic backgrounds) was prevented by acute inhibition of ROCK2. In a previous study, SLX‐2119 did not affect endothelial function (or vasodilation to nitroprusside) in carotid arteries from male C57BL/6 mice, but did restore responses to acetylcholine in vessels treated with an activator of RhoA or Ang II (De Silva et al., 2016). Here we extend those findings to both sexes as well as mice on a second genetic background.

We have previously found that Ang II‐induced endothelial dysfunction in both the incubation model and in vivo is mediated via the AT1R (Li et al., 2016; Ryan et al., 2004). Because we used both carotid arteries for studies of vasomotor function, we did not have segments of these vessels left for quantification of ROCK protein or mRNA. Since our data suggested that both effects of Ang II and inhibition of ROCK2 were similar in male and female mice as well as mice with different genetic backgrounds, we did not feel there was a strong rationale to expect that differences in expression would account for the similarities between the groups. Thus, using additional groups of mice and other resources for expression assays did not seem well justified. In addition, previous studies from our laboratory and others suggest vascular expression of ROCK isoforms often does not change in models of disease (De Silva et al., 2015; Nuno et al., 2012). We recognize that the lack of data on possible changes in vascular expression represents a limitation of this study.

### 4.5 Implications

Endothelial dysfunction is a cornerstone event in mechanisms that contribute to the initiation and progression of vascular disease, including atherosclerosis. The RAS and specifically Ang II are important drivers of atherosclerosis (Daugherty et al., 2004; Hu et al., 2017). In preclinical studies, limited data exists in relation to carotid artery disease, the presence or absence of sex‐dependent differences, or the impact of genetic background. In relation to mechanisms that contribute to loss of endothelial function, numerous studies suggest that oxidative stress plays a key role, but the impact of ROCK and specific ROCK isoforms is poorly defined. In this context, our findings provide the first evidence that ROCK2 may be a key contributor to endothelial dysfunction in both sexes and in genetic strains that differ in relation to other major aspects of vascular disease. Overall, our experiments provide new insight into phenotypic effects of Ang II on arteries and the mechanistic impact of ROCK2.

## CONFLICT OF INTEREST

None.

## ETHICAL STATEMENT

The authors declare no conflicts of interest.
